# Significance of human epidermal growth factor receptor 2 expression in colorectal cancer

**DOI:** 10.3892/etm.2014.2063

**Published:** 2014-11-11

**Authors:** JINHUA TU, YINGHAO YU, WEI LIU, SHUNPING CHEN

**Affiliations:** 1 Department of Pathology, The First Affiliated Hospital of Xiamen University, Xiamen, Fujian 361004, P.R. China; 2 Department of Pathology, Dongfang Hospital, Fujian Medical University, Fuzhou, Fujian 350025, P.R. China

**Keywords:** colorectal cancer, human epidermal growth factor receptor 2, immunohistochemistry, fluorescence *in situ* hybridization, herceptin

## Abstract

The aim of the present study was to evaluate the protein expression level of human epidermal growth factor receptor 2 (HER-2) using immunohistochemistry (IHC), and assess the association with clinicopathological parameters and the prognosis of patients with colorectal cancer (CRC). In addition, the current study observed the consistency between the levels of HER-2 protein expression determined by IHC and HER-2 gene amplification determined by fluorescence *in situ* hybridization (FISH) in the CRC samples. Overexpression of HER-2 and gene amplification were examined with semiquantitative standardized IHC in 878 formalin-fixed paraffin-embedded CRC samples, while 102 of these cases were analyzed with FISH. A total of 102 cases (11.6%), out of the 878 cases, were determined by IHC to overexpress HER-2. Of these, 25 cases were strongly positive (IHC3+), while 77 cases revealed moderate staining (IHC2+). HER-2 overexpression was more frequent in early-stage cases compared with advanced-stage cases of CRC (P<0.001). However, there was no association observed between HER-2 overexpression and clinicopathological parameters. FISH analysis revealed that 64% (16/25) of the IHC3+ cases had HER-2 gene amplification. By contrast, only 6.5% (5/77) of the IHC2+ cases, and none of the 20 randomly selected IHC0 or 1+ cases, demonstrated HER-2 gene amplification. Furthermore, no associations were observed between HER-2 overexpression or gene amplification with the survival time. Thus, the present study observed that HER-2 overexpression does not correlate with other clinicopathological data or the survival rate, with the exception of clinical stages. However, IHC3+ and 2+ cases should be further analyzed by FISH to assess the status of the HER-2 gene in CRC. Patients with HER-2 gene amplification may constitute as potential candidates for targeted therapy with trastuzumab.

## Introduction

Colorectal cancer (CRC) is one of the most common types of tumor and is the fourth leading cause of cancer mortality worldwide (1). With economic development and changes in lifestyle, the incidence and mortality rates of CRC have rapidly increased in China. With the exception of surgery, the majority of treatments for CRC, including traditional chemo- and radiation therapies, are not fully efficacious in treating the disease. The recent development of novel drugs targeting CRC has improved the survival rate of patients with the disease. Targeted drugs for the treatment of cancer have rapidly developed. The human epidermal growth factor receptor 2 (HER-2) signaling pathway plays an important role in tumor proliferation, angiogenesis, differentiation and metastasis in CRC (2). HER-2-targeted drugs, including Herceptin, have been developed and widely applied for the treatment of breast cancer presenting with membranous HER-2 overexpression (3,4).

The HER-2 oncogene is a member of the tyrosine kinase family of receptors, which includes HER-1, also known as epidermal growth factor receptor (EGFR), HER-2, HER-3 and HER-4. HER-2 is located on chromosome 17q21 and encodes a 185 kDa transmembrane protein. HER-2 activation initiates signal cascades, including the mitogen-activated protein kinase and phosphoinositide 3-kinase/Akt signaling pathways, which are essential for cell proliferation and differentiation. Thus, HER-2 overexpression leads to the disordered proliferation and malignant transformation of cells. HER-2 is overexpressed in numerous types of malignant cancer, including breast, ovarian, gastric, lung, colorectal and prostate cancers (5). Chen *et al* (6) revealed that the detection of HER-2 protein expression may be used to assess the malignant biological behavior and prognosis of gastric cancer. The European Committee has already approved chemotherapy combined with Herceptin as a treatment for cases of gastric cancer presenting with membranous HER-2 overexpression (7). Thus, ensuring that the expression level of HER-2 in patients with gastric cancer is examined accurately is of importance. However, conflicting data exist with regard to the prevalence of HER-2 overexpression in CRC, with a range between 2 and 47%, while the prevalence of HER-2 gene amplification ranges between 2.5 and 7.4% (8–27). Similarly, there is controversy in the published literature with regard to the association between the survival rate and HER-2 overexpression in CRC (10–13,28).

Since there are few published studies investigating HER-2 expression in CRC and genetic differences exist between ethnic groups with regard to tumorigenesis, numerous topics require further study. Thus, the present study investigated the frequency of HER-2 overexpression and gene amplification in CRC, and whether HER-2 overexpression and gene amplification were consistent. In addition, associations between HER-2 overexpression with clinicopathological parameters and the prognosis of CRC were analyzed.

## Materials and methods

### Patients and tissue specimens

Clinicopathological data and paraffin-embedded specimens were collected from 878 patients who underwent colorectal resections at Dongfang Hospital (Fuzhou, China) between January 2006 and April 2012. The study was approved by the ethics committee of Dongfang Hospital and written informed patient consent was obtained from the patient or the patient’s family. Of the 878 patients, 541 were male and 337 were female, with ages ranging between 17 and 85 years (median age, 51 years). A total of 396 tumors were located in the rectum, while 482 tumors were in the colon. None of the patients had received preoperative neoadjuvant chemotherapy or radiotherapy. A total of 100 paraneoplastic normal tissue specimens of CRC were used as controls.

The conditions of the patients were assessed according to the Tumor Node and Metastasis (TNM) Classification of Malignant Tumors (29). TNM classification revealed that 490 (55.8%) patients were at stages 0, I or II, while 388 (44.2%) patients were at stages III or IV. One tumor was classified as pTis, 148 tumors were pT1, 341 tumors were pT2, 314 tumors were pT3 and 74 tumors were pT4. The World Health Organization Classification of Tumors was used for histological classification (30). A total of 761 (86.7%) tumors were classified as well and moderately differentiated, while 117 (13.3%) were poorly differentiated. All the specimens were routinely fixed in 10% formalin, embedded in paraffin and verified pathologically prior to inclusion in the present study. Follow-up was conducted at 6, 12, 18 and 24 months following surgery and in one-year intervals thereafter. Patients who succumbed within four weeks following radical surgery and those who were >85-years-old were excluded from the current analysis.

### Immunohistochemical staining

HER-2 overexpression analysis was conducted on 4-μm-thick sections. Briefly, following deparaffinization and rehydration, the tissue samples were incubated in a citrate buffer solution at 90–95°C for 20 min. The slides were washed with phosphate-buffered saline (PBS) for three times for 3 min. Endogenous peroxidase activity was suppressed by a 10 min incubation in methanol with 3% hydrogen peroxide. A primary monoclonal rabbit antibody against the human HER-2 protein (Clone SP3; Lab Vision Corporation, Fremont, CA, USA) was applied for 60 min at room temperature. Subsequently, a secondary goat anti-rabbit antibody (Lab Vision Corporation) conjugated to horseradish peroxidase was applied for 30 min at room temperature. The bound antibody was visualized using a peroxidase chromogen substrate. The sections were counterstained with hematoxylin and a coverslip was applied. Paraffin slides of invasive breast carcinomas were used as a positive control (these were obtained from the Department of Pathology, Dongfang Hospital). For antibody-negative controls, the primary antibodies were substituted with PBS.

Slides were examined separately by two independent pathologists blinded to each others results. Discrepancies between the investigators (<5% of cases) required a third joint observation with a conclusive agreement. The HercepTest^™^ scoring system specific to gastric cancer was used to determine tumor cell reactivity, as described by Hofmann *et al* (31) in 2008. No reactivity or membranous reactivity in <10% of the tumor cells was defined as an immunohistochemistry (IHC) score of 0; faint/barely perceptible partial membrane reactivity in >10% of the tumor cells was defined as a score of 1+; weak to moderate complete or basolateral membranous reactivity in >10% of the tumor cells was defined as a score of 2+; strong complete or basolateral membranous reactivity in >10% of the tumor cells was defined as a score of 3+. A score of 0 or 1+ was considered negative, while a score of 2+ or 3+ was considered positive. Cytoplasmic staining may have been present, but was not included in the determination of positivity.

### Fluorescence in situ hybridization (FISH)

FISH analysis was applied to all IHC2+ and 3+ tumors, as well as to 20 randomly selected IHC0 and 1+ cases. Paraffin slides of invasive breast cancers were used as a positive control. FISH was conducted with a HER-2 DNA Probe kit (GP Medical Technologies, Ltd., Beijing, China), according to the manufacturer’s instructions. The commercially available double-color FISH probe consisted of two probes: 17q11.2-q12 (labeled with a red signal) covering the whole HER-2 gene and the control, centromeric chromosome 17p11.1-q11.1 (labeled with a green signal).

The FISH-fixed glass slides with 4-μm-thick sections were heated overnight at 65°C, deparaffinized in two 10-min changes of xylene, rehydrated with two 3-min changes of 100% ethanol, one 3 min change of 85% ethanol and one 3 min change of 70% ethanol, and immersed for 15 min in pure water at 90°C. The slides were incubated (in a water bath) for 35 min in sodium sulfite acid at 50°C and washed in 2X sodium saline citrate (SSC; pH 7.2) at room temperature. The slides were incubated for 12 min in proteinase K solution at 37°C, washed in 2X SSC (pH 7.2) at room temperature, dehydrated with 70, 85 and 100% ethanol and allowed to air-dry. To denature the DNA, the slides were placed in 78.5°C preheated 70% formamide/2X SSC for 8 min and dehydrated in a graded series of ethanol concentrations that had been precooled to −20°C. Following air-drying, 10 μl probe, which had been previously destructured at 75.5°C for 7 min, was applied onto each slide. A cover slip was placed and sealed with rubber cement, and the slides were hybridized at 42.8°C overnight. After 16–18 h hybridization, the slides were washed in 46°C preheated post-hybridization buffer (2X SSC/0.1% sodium dodecyl sulfate) for 5 min and rinsed in 70% ethanol. Following air-drying (out of direct light), the slides were counterstained with 10 μl 4′,6-diamidino-2-phenylindole/anti-fade solution and coverslips were applied.

After 10 min, the slides were observed under a fluorescence microscope (Olympus BX51; Olympus, Tokyo, Japan). A total of 30 randomly selected tumor nuclei were evaluated in three separate, distinct microscopic areas. Cases were classified as negative/no amplification when the HER-2 gene (red signal) to centromeric probe 17 (green signal) ratio was <1.8, while cases with a ratio of >2.2 were classified as positive/amplification. When the ratio was between 1.8 and 2.2, ≥100 randomly selected tumor nuclei were evaluated. Furthermore, a cell was considered to demonstrate amplification when a definite cluster or >10 red signals for the HER-2 gene were identified, as described in a previous FISH study (32). Cases were defined as chromosome 17 polysomy when the green signal was >2.25 in each nucleus when counting ≥30 tumor nuclei.

### Statistical analysis

The χ^2^test was performed to analyze the association between HER-2 overexpression and the clinicopathological characteristics of CRC, and the correlation between IHC and FISH. The probability of survival for the various subgroups was calculated using the Kaplan-Meier method. All statistical analyses were performed two-sided, where P<0.05 was considered to indicate a statistically significant difference. SPSS 16.0 software (SPSS, Inc., Chicago, IL, USA) was used for analysis.

## Results

### Overexpression of HER-2

**Figure 1:**
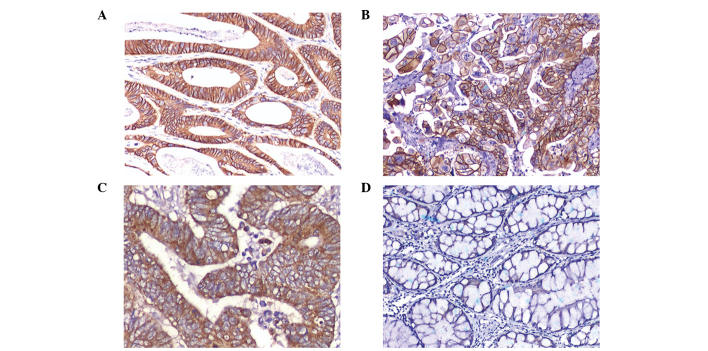
Immunohistochemical staining for human epidermal growth factor receptor 2 (HER-2). (A) HER-2 (IHC3+), well differentiated colorectal cancer (CRC; magnification, ×200). (B) HER-2 (IHC3+), moderately and poorly differentiated CRC (magnification, ×200). (C) HER-2 (IHC2+), well and moderately differentiated CRC (magnification, ×200). (D) HER-2 was negative in normal epithelial cells adjacent to the tumor tissue (magnification, ×100).

A total of 102 cases (11.6%) out of the 878 patients were demonstrated to have overexpressed HER-2 by IHC. Of these, 25 cases were strongly positive (3+; Figs. 1A and B) and 77 cases revealed moderate staining (2+; Fig. 1C). HER-2 overexpression was more frequent in 0, I and II stage cases compared with stage III and IV cases (P<0.001). No association was observed between HER-2 overexpression and gender, age, tumor site, size, depth of invasion, lymph node metastases or distant metastases (P>0.05; Table I). Stromal and normal epithelial cells adjacent to the tumor tissue were negative (Fig. 1D).

### HER-2 gene amplification

**Figure 2:**
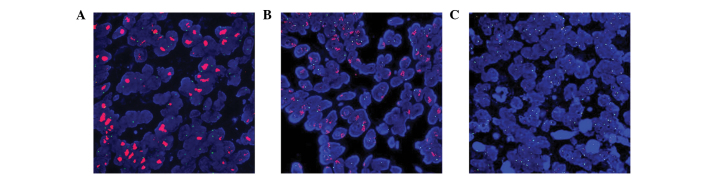
Fluorescence *in situ* hybridization analysis of human epidermal growth factor receptor 2 (HER-2) gene amplification (magnification, ×1,000). (A) Positive amplification of the HER-2 gene, as indicated by red cluster signals in the tumor cells. (B) Positive amplification of the HER-2 gene; the ratio of the HER-2 gene (red signals) to centromeric probe 17 (green signals) was >2.2. (C) Negative amplification of the HER-2 gene; the ratio of the HER-2 gene (red signals) to CEP 17 (green signals) was <1.8.

Following FISH analysis, 24.5% (25/102) of the IHC3+ cases were shown to exhibit HER-2 gene amplification (Fig. 2A). By contrast, only 6.5% (5/77) of IHC2+ cases (Fig. 2B), and none of the randomly selected 20 cases with IHC0/1+, demonstrated HER-2 gene amplification (Fig. 2C). A relatively high level of consistency was observed between IHC3+ and IHC0/1+ with FISH (64 and 100%, respectively); however, there was a low level of consistency with the results between IHC2+ and FISH (6.5%; Table II).

### Chromosome 17 polysomy and non-polysomy

Chromosome 17 copy number analysis was applied to all IHC2+ and 3+ cases. Two cases (8%) revealed chromosome 17 polysomy out of 25 IHC3+ cases, while only one case (1%) was identified in the 77 IHC2+ cases. Among the 21 tumors with HER-2 gene amplification, only one case (5%) exhibited chromosome 17 polysomy, while two cases (2.5%) were observed in the 81 cases without HER-2 gene amplification. In the FISH-positive cases, there was one case (6.3%) of chromosome 17 polysomy in 16 IHC3+ cases and no chromosome 17 polysomy observed in the five IHC2+ cases. With regard to the FISH-negative cases, one case (11%) out of nine IHC3+ cases and one case (1%) out of the 72 IHC2+ cases had chromosome 17 polysomy (Table III).

### Survival analysis

**Figure 3:**
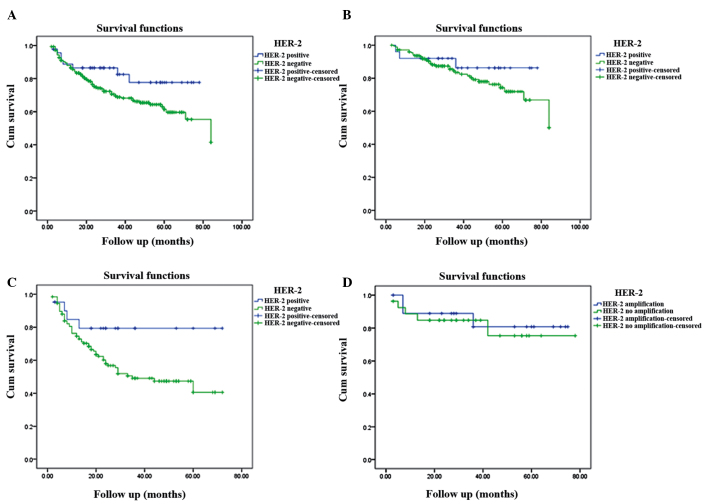
Kaplan-Meier survival analysis comparing HER-2 positivity [immunohistochemistry (IHC)3+ and 2+] with HER-2 negativity in (A) colorectal cancer (CRC; P=0.082), (B) early-stage CRC (P=0.328) and (C) advanced-stage CRC (P=0.06). (D) Kaplan-Meier survival analysis comparing HER-2 amplification with HER-2 non-amplification in HER-2-positive cases (P=0.736). Cum, cumulative; HER-2, human epidermal growth factor receptor 2.

Follow-up was conducted on 349 cases, including 46 HER-2-positive (IHC3+ and 2+) and 303 HER-2-negative (IHC0 and 1+) cases. Among the 349 cases, 202 were early-stage (0, I or II stage; HER-2-positive, 25; HER-2-negative, 177) and 147 were advanced stage cases (III or IV stage; HER-2-positive, 21; HER-2-negative, 126). The median follow-up duration was 28 months (range, 2–84 months) and 135 cases were followed for >3 years. The median survival time was 84 months and the mean survival time was 60.9 months. The mean survival times of the HER-2-positive and -negative groups were 64.9 and 59.5 months, respectively. The HER-2-positive CRC patients exhibited higher three- and five-year survival rates compared with HER-2-negative patients (77.7 vs. 68.8% and 77.7 vs. 61.4%, respectively); however, the difference was not statistically significant (P=0.082; Fig. 3). HER-2-positive patients with early and advanced stage CRC revealed higher survival rates compared with HER-2-negative cases at three years (86.2 vs. 83.5% and 79.4 vs. 49.1%, respectively) and five years (86.2 vs. 74.3% and 79.4 vs. 40.6%, respectively); however, this difference was also not statistically significant (P=0.328 and P=0.06, respectively; Fig 3). A total of 20 HER-2 gene amplification and 26 gene non-amplification cases were included in the 46 HER-2-positive cases. In the HER-2-positive group, HER-2 gene amplification and non-amplification exhibited a three-year survival rate of 80.8 vs. 84.7%, respectively, and five-year survival rate of 80.8 vs. 75.3%, respectively (Fig 3); however, this difference was not statistically significant (P=0.736). In general, there was no association between HER-2 overexpression or gene amplification and survival time.

## Discussion

In the present study, HER-2 overexpression was observed in 102 (11.6%) of the 878 Chinese CRC samples. Previous studies have reported positive rates of HER-2 overexpression in CRC ranging between 2 and 47.4%. The positive rates of HER-2 overexpression may have varied in these studies due to differences in the IHC procedure, sample size and the scoring system employed. Park *et al* (10) revealed HER-2 overexpression in 47.4% of 137 patients with CRC, whereas Antonacopoulou *et al* (16) observed overexpression in 24.7% of 124 patients using IHC performed on whole sections. Demirbas *et al* (11) demonstrated HER-2 overexpression in 9.6% of 104 patients with CRC using tissue microarray (TMA). The results of these studies indicate that the expression of HER-2 in CRC is associated with the prognosis and may constitute a potential candidate for novel adjuvant therapies involving humanized monoclonal antibodies, such as Herceptin. However, other studies have demonstrated that the expression of HER-2 in CRC was not associated with the prognosis, based on a subjunctive scoring system of IHC. Kruszewski *et al* (20) reported HER-2 overexpression in 27% of 202 CRC patients, while Kavanagh *et al* (12) observed overexpression in 11% of 132 patients using IHC performed on whole sections. Kim *et al* (23) reported HER-2 overexpression in 0.5% of 185 patients with CRC, and Marx *et al* (24) reported overexpression in 2.7% of 1,851 patients using TMA. Furthermore, a number of studies have demonstrated that HER-2 overexpression was not associated with gender, age, histological tumor type, tumor localization, grading, pT, pN, pM or survival (12,22,28).

Consequently, there are two hypotheses on the role of HER-2 expression in CRC at present. Firstly, HER-2 overexpression may be an independent prognostic factor in CRC, whilst secondly, expression of HER-2 in CRC is not associated with prognosis. No associations between HER-2 overexpression and gender, age, tumor site, size, depth of invasion, lymph node metastases or distant metastases (P>0.05) were observed in the present study. Furthermore, no statistically significant difference was observed between HER-2 amplification and HER-2 non-amplification (P=0.736) in the three- and five-year survival rates. Thus, the current data were consistent with the latter hypothesis that HER-2 overexpression is not an independent prognostic factor of CRC. However, the present study also revealed that HER-2 overexpression was associated with the TNM stage. Early-stage cancers exhibited a higher rate of HER-2 overexpression compared with advanced-stage cancers (16.1 vs. 5.9%; P<0.001). However, this observation is not consistent with those of previous studies where the HER-2 positivity rate of early-stage cancers was lower than that of advanced-stage tumors (16,33), or where the HER-2 positivity rate of cancers was shown not be associated with the TNM stage (12,20,24,26,34).

A previous study demonstrated that Herceptin, an anti-HER-2 monoclonal antibody, inhibits HCA-7 cell proliferation *in vitro* and *in vivo* (35). As the first HER-2 dimerization inhibitor, pertuzumab (a monoclonal antibody), also exhibits antitumor activity on human colon cancer cells *in vitro* and *in vivo*, in particular when combined with erlotinib (36). A phase II trial revealed that a low overexpression rate of HER-2 (8.0%) in advanced CRC limits the application of Herceptin as a treatment for advanced-stage CRC; however, partial responses were observed in five of the seven evaluable patients (17). Annually, there are ~one million new cases of CRC worldwide, indicating that of these, 100,000 cases may overexpress HER-2, according to the HER-2 positivity rate of 11.6% in the present study. HER-2 gene amplification is vital for targeted tumor therapy, such as Herceptin for breast tumors. However, not all HER-2-positive cases exhibit HER-2 gene amplification.

In the current study, HER-2 gene amplification was observed in 21% (21/102) of the tumors exhibiting HER-2 overexpression and in 2.4% of the total 878 cases of CRC. These results were similar to those from previous studies where the HER-2 gene amplification rate ranged between 2.5 and 7.4% (11,19,24). Liu *et al* (37) reported that the rate of consistency between IHC and FISH was 70% for IHC3+ and 14% for IHC2+ in gastric cancer samples. In the present study, a relatively high consistency rate was observed between IHC3+ and IHC0/1+ with FISH (64 and 100%, respectively); however, there was a low consistency result between IHC2+ and FISH (6.5%). Thus, the concordance rate between IHC and FISH in CRC is analogous to that observed in gastric cancer.

A number of studies have demonstrated that chromosome 17 polysomy may be the main reason for HER-2 overexpression but not HER-2 gene amplification (37–39). In the present study, only one case (11%) of chromosome 17 polysomy was observed out of nine IHC3+ cases with no HER-2 gene amplification. In addition, there was only one case of chromosome 17 polysomy in the 72 IHC2+ cases with no HER-2 gene amplification. Thus, it was hypothesized that chromosome 17 polysomy may not be the reason for HER-2 positivity without HER-2 gene amplification in CRC. It may be that different mechanisms result in HER-2 overexpression, including transcriptional activation by other genes, post-transcriptional events or a new genomic environment associated with amplification (40–42). However, it is considered that the inconsistency between HER-2 overexpression and gene amplification is associated with the two methods, namely of immunohistochemical staining and fluorescence *in situ* hybridization.

IHC is less expensive and time-consuming, easy to store and perform, and requires a routinely available microscope. However, the IHC techniques may be potentially affected by a number of variables, including tissue fixation, processing, primary antibody selection, detection systems and methods of antigen retrieval. Furthermore, as the proposed scoring system for IHC is subjective, interpretation may vary among observers. These factors, in addition to the small study sample sizes, may also account for the variable rates of HER-2 immunoreactivity, as well as the conflicting results indicating that HER-2 is associated with adverse clinical outcomes in certain studies, but not in others. At present, FISH is regarded as the most effective method for the detection of HER-2 amplification, as it is has high rates of sensitivity and specificity. FISH is also advantageous as it can be conducted with small tumor samples and with formalin-fixed and paraffin-embedded tissue samples. This technique allows for the direct visualization of gene amplification in the nuclei and provides an objective count of genes and chromosomes on a cell-by-cell basis. However, this method is expensive, time-consuming, requires a fluorescent microscope and is difficult to separate *in situ* and invasive carcinomas. Furthermore, fluorescence fades rapidly; thus, a permanent record is not created (32,43,44).

CRC involves changes in multiple oncogenes, tumor suppressor genes and signal transduction pathways. Almost all tumors with more than one locus are involved in tumorigenesis. EGFR inhibitors have been widely used in oncotherapy. The identification of the mutant kirsten rat sarcoma viral oncogene homolog (KRAS) as a predictor of resistance to EGFR monoclonal antibodies created a major change in the treatment of CRC (43–45). As it is known drug resistance results in the failure of chemotherapy and poor prognosis. However, it also remains a cause for limiting the EGFR inhibitor long-term efficacy, with the exception of the KRAS mutant that plays a vital role in predicting EGFR monoclonal antibodies in CRC. EGFR inhibitor resistance is associated with the mechanisms that follow signal pathway activation of HER-2, VEGF and platelet-derived growth factor. In other words, all of these are activated by circumventing EGFR protein tyrosine kinase signaling pathway, activation. Herreros-Villanueva *et al* (25) hypothesized that HER-2 gene amplification may be one of the causes of insensitivity to anti-EGFR therapies, including cetuximab. The study reported that HER-2 gene amplification was observed in 26.3% of KRAS and v-raf murine sarcoma viral oncogene homolog B (BRAF) wild type colorectal carcinomas in Spanish patients. In addition, previous studies have reported that KRAS and BRAF mutations are mutually exclusive in CRC; if there are KRAS mutations, no BRAF mutations are present, and vice versa (45,46). The KRAS mutation statuses in 280 samples selected from 878 patients with CRC were detected in a previous study (47). The results revealed that there were no cases of HER-2 gene amplification in KRAS mutant types, with all HER-2 gene amplification occurring in the KRAS wild type. Considering that there were no cases of the KRAS mutation type in the patients with CRC, whether KRAS mutations and HER-2 gene amplification are mutually exclusive remains to be elucidated. Furthermore, the association between BRAF mutations and HER-2 gene amplification requires further investigation, as well as whether HER-2 monoclonal antibodies may be used to aid EGFR inhibitor resistance in CRC.

As the standard treatment for breast and gastric cancers, the premise of the success of Herceptin is concordance between HER-2 expression and gene amplification. A previous study revealed that lapatinib, an EGFR/HER-2 kinase inhibitor, combined with Panobinostat, a histone deacetylase inhibitor, interacted synergistically to inhibit the proliferation and colony formation in all CRC cell lines tested. Compared with either agent alone, there was no apparent increase in toxicity (48). A phase II trial revealed that Herceptin exerted a therapeutic effect on CRC, although the low overexpression rate of HER-2 (8.0%) in advanced CRC limited the efficacy of the drug (17). Chen *et al* (49) revealed that there was no statistically significant difference in HER-2 expression between colorectal liver metastases and the corresponding primary tumors. Thus, metastatic lesions may also be suitable for anti-HER-2 therapy due to the homogenicity of HER-2 expression in CRC. These results indicate that HER-2 may be a promising target as an adjuvant therapy for patients with CRC. However, to determine the precise curative effect of anti-HER-2 therapy on CRC, multicenter or international cooperation is required, through large clinical trials, to study the association between the HER-2 and KRAS genes.

In conclusion, HER-2 overexpression and gene amplification are present in CRC. With the exception of clinical stages, no associations were observed between HER-2 overexpression and other clinicopathological data in the present study. HER-2 overexpression and gene amplification did not correlate with established prognostic indicators. However, IHC3+ and 2+ cases should be further analyzed by FISH to assess the gene status of HER-2 in CRC. Patients with HER-2 gene amplification may be potential candidates for targeted therapy with Herceptin. However, further studies are required to confirm these results.

## Figures and Tables

**Table I:** Association between HER-2 overexpression (IHC3+ and 2+) and the clinicopathological characteristics of CRC.

Clinicopathological characteristic	Cases, n	HER-2 overexpression, n (%)	P-value
Gender			
Male	541	64 (11.8)	0.803
Female	337	38 (11.3)	
Age, years			
<60	443	51 (11.5)	0.922
≥60	435	51 (11.7)	
Tumor site			
Colon	482	50 (10.4)	0.204
Rectum	396	52 (13.1)	
Tumor size, cm			
<5	445	49 (11.0)	0.570
≥5	433	53 (12.2)	
Depth of invasion			
Tis+T1	12	0 (0.0)	0.514
T2	174	20 (11.5)	
T3	648	79 (12.2)	
T4	44	3 (6.8)	
Classification			
Well and moderate	761	94 (12.4)	0.083
Moderate and poor	117	8 (6.8)	
TNM stage			
0/I/II	490	79 (16.1)	0.000
III/IV	388	23 (5.9)	
Lymph node metastases			
N0	513	56 (10.9)	0.611
N1	229	27 (11.8)	
N2	136	19 (14.0)	
Distant metastases			
M0	804	92 (11.4)	0.595
M1	74	10 (13.5)	

TNM, tumor node and metastsis; HER-2, human epidermal growth factor receptor 2; IHC, immunohistochemsistry; CRC, colorectal cancer.

**Table II:** Concordance analysis between HER-2 overexpression and amplification.

		FISH, n (%)		
			
IHC status	Cases, n	Amplification	No amplification	Concordance (%)
IHC3+	25	16 (64.0)	9 (36.0)	64.0
IHC2+	77	5 (6.5)	72 (93.5)	6.5
IHC0/1+	20	0 (0.0)	20 (100.0)	0.0
Positive control	10	10 (100.0)	10 (0.0)	100.0

IHC, immunohistochemistry; FISH, fluorescence *in situ* hybridization; HER-2, human epidermal growth factor receptor 2.

**Table III:** Association between chromosome 17 copy number and HER-2 overexpression/amplification.

IHC/FISH status	Cases, n	Chromosome 17 copy number, n (%)	

		Polysomy	Non-polysomy
IHC3+	25	2 (8)	23 (92)
IHC2+	77	1 (1)	76 (99)
FISH+	21	1 (4.8)	20 (95.2)
FISH-	81	2 (2.5)	79 (97.5)
IHC3+/FISH+	16	1 (6.3)	15 (93.7)
IHC2+/FISH+	5	0 (0)	0 (100)
IHC3+/FISH-	9	1 (11)	8 (89)
IHC2+ /FISH-	72	1 (1)	71 (99)

IHC, immunohistochemistry; FISH, fluorescence *in situ* hybridization; HER-2, human epidermal growth factor receptor 2.
